# Dr. Elliotson on the Recent Improvement in the Art of Distinguishing Diseases of the Heart

**Published:** 1831-01-01

**Authors:** 


### On the recent Improvements in the Art of distinguishing the various Diseases of the Heart, &c. By *John Elliotson*, M.D.

### Lecture II.—*Affections of the Lining Membrane of the Heart.*

The lining membrane of the heart's cavities and valves is occasionally found intensely red, the deep colour generally extending to the aorta or pulmonary artery, and not to be washed away by water, except by maceration of several days' continuance. This cannot always, Dr. E. thinks, be ascribed to the imbibition of blood; because frequently no blood is found in contact with the reddened part—and the artificial application of blood to the interior of an artery during 48 hours, has failed to redden it to the same degree.

"I have seen the heart and aorta quite empty where this redness existed. Neither can it be ascribed to congestion from dyspnœa, for I have seen it where death had taken place in a moment in the midst of perfect general health; nor to decomposition, for I have witnessed it on the day following death in cold weather. I have seen it intense, even when all these conditions were united—fixedness of colour, freedom from the contact of blood, sudden death, perfect general health, and absence of decomposition. Of course, mechanical obstruction to the passage of the blood from the vascular system of the heart, or a softened state of the organ, either by disease or by incipient decomposition, will cause the blood in the substance or the cavities of the heart to redden the lining membrane deeply." 13.

Seeing, then, that these crimson hues of the heart's linings have been found under all kind of circumstances, and where the individual died suddenly in perfect health, we cannot help suspecting that, in all cases where we merely find the red tinge, without any other of the physical characters of inflammation, as injection of the vessels, thickening of the membranes, effusions of lymph, &c. the said tinge is to be attributed rather to imbibition than previous disease.

Dr. E. is not aware of any cardiac symptoms to which this inflammation could give rise, except rapidity of pulse—increased force and sound of the heart's action, exactly as occurs in pericarditis. When, indeed, the portion of membrane lining the valves is not only inflamed, but thickened and bound down by fibrinous exudations, we have the bruit de scie or the bruit de soufflet. If this sound be absent, Dr. E. presumes that the acute inflammation of the lining membrane can be distinguished from pericarditis, "by only the absence of pain in the particular directions mentioned in the last lecture, and of tenderness on pressure applied in the manner pointed out."

"I have never opened a person who died with this acute affection; but four times I have suspected its existence when acute pericarditis was present. The only ground of my suspicions was the occurrence in these cases of a sound like that of a bellows with the contraction of the heart,—a symptom which the most assiduous French auscultators, Laennec, Bertin, Andral, Collin, do not consider as belonging to pericarditis, and which the experience of all my friends, accustomed, like myself, to employ the stethoscope in every case of pectoral disease, as well as my own, shows to be no symptom of mere pericarditis. Indeed there is an impossibility in imagining how it should, for we hear it every day where no pericarditis exists, from any mechanical disturbance to the course of the blood in any point of its progress through the heart, or even through the arteries. In two of these four cases of acute pericarditis, in which I heard it, the symptoms of pericarditis were very mild; yet the sound was considerable, and it ceased after antiphlogistic measures had been employed. On the other hand, I have attended numerous cases of very violent and well-marked cases of acute pericarditis, and not heard it, though in none did I ever omit daily to employ the stethoscope, and, as well as some of my friends, have made this a point of careful investigation. In the two other cases, the symptoms of pericarditis were certainly violent, but, after they were entirely subdued, the bellows-sound continued as loud as before: evidently, therefore, not the result of the presence of pericarditis, but of those organic changes that every day give rise to the bellows-sound, and are often preceded by pericarditis, as I mentioned in the last lecture. For the same reason, I have known it not begin till all the symptoms of the pericarditis had actually subsided.

In acuto-chronic and chronic pericarditis this sound is not uncommon; and, when once it has begun, it may subside after some weeks, or continue till death, lasting, perhaps, for years; and depends not upon the pericarditis, for this may at length cease and possibly the whole membrane concrete, but almost always upon chronic disease of the lining membrane about the valves and openings, or some induration or enlargement external to the openings or passages from the heart and compressing them, or upon dilation of a cavity behind an opening.

I have never opened a person whom I had seen labouring under chronic pericarditis and affording the bellows-sound *constantly*, in whom a diseased condition of an opening or passage from the heart did not present itself—in whom the opening or passage was not below its natural proportion to the cavity behind it, being itself lessened or compressed, or the cavity behind it increased, so that it was become virtually too small. The occasional simultaneous occurrence of acute inflammation of the lining membrane or substance of the heart, with *acute* inflammation of the pericardium, effusing lymph about the openings, inducing spasmodic constriction of them, or turgescence around them; and the tendency of the lining membrane and substance of the heart to become diseased, and of morbid depositions to take place externally to the roots of the two great arteries, when the pericardium is the seat of *chronic* inflammation,—are the reasons, I am satisfied, that the bellows-sound is ever heard in conjunction with the symptoms of pericarditis." 14.

The lining membranes about the boundaries of the cardiac cavities—like those about the parts of other cavities, are the spots most liable to be affected. Scirrhus and stricture of the alimentary canal chiefly affect the cardia, the pylorus, and the rectum—and ulceration in fever generally seizes on the termination of the ileum. The larynx and air-cells are the parts of the respiratory tubes which are mostly affected with ulceration—the lips are frequently affected with cancer—the cervix uteri and neck of the bladder with organic disease—and so inflammatory thickening, and all other diseases of the lining membrane of the heart, far most frequently occupy that portion which invests the openings, and assists to form the valves. We offer the following passage without comment, as it bears on a fiercely mooted point of physiology at the present moment.

"Perhaps I may be permitted to say a few words on the natural sounds of the heart's action. Besides the impulse which is given by the heart to the left side of the chest, its action affords a distinct sound in health on applying the ear, either immediately to the parietes, or with the intervention of a solid medium. This sound is double, and indeed in many persons perceptibly so to themselves on lying upon the left side. The first part is heard at the moment the pulse is felt in the wrist or after the smallest possible interval: the second immediately afterwards. The first, according to Laennec, arises from the ventricular action; the second from the auricular. The first is rather dull and long; the second short and clear—a kind of smack. After the second, a short pause ensues, before the dull sound of the ventricles, the stroke of the heart's apex, and the pulse at the wrist, recur. The whole period is estimated by Laennec at about one-half for the ventricular contraction, one-quarter for the auricular, and one-quarter for the pause. The sound of the auricles is heard best at the upper part; of the ventricles, at the lower part, of the cardiac region. The sounds of the left auricle and ventricle are heard best at the cartilages of the fifth, sixth, and seventh left ribs; those of the right auricle and ventricle, at the sternum.

When the passage of the blood from either ventricle is materially impeded, the sound which is synchronous with the pulse, and loudest at the lower part of the cardiac region, is changed to the blowing, whizzing shrill, or cooing character just mentioned; if the impediment is in the left ventricle—at the mouth of the aorta, it is loudest at the cartilages of the ribs to the left of the sternum; if in the right ventricle—at the mouth of the pulmonary artery, it is loudest at the sternum and to the right. The sound is often so loud, that it prevents the natural sound of the auricles from being distinctly perceptible till the ear or stethoscope is removed from the region of the ventricles higher,—to the region of the auricles.

When the impediment is at either of the auriculo-ventricular openings, the morbid sound is heard at the moment of the auricular contraction,—not synchronously with the radial pulse and the stroke of the heart's apex; and is generally loudest at the superior part of the cardiac region. It is loudest at the cartilages of the left ribs, when the left auriculo-ventricular opening is narrowed; loudest at the sternum and to the right, when the narrowing is at the right auriculo-ventricular opening. When an auriculo-ventricular opening is narrowed, and the morbid sound is heard at the auricular contraction, the action of the auricle is not instantaneous as in health, but rather slower, so that we may fancy we hear the cavity gradually empty itself. I do not think this slowness so observable in cases of narrowing of the mouth of the pulmonary artery or aorta.

Laennec's correctness in ascribing the first of the two sounds of the heart's action in health to the ventricle and the second to the auricle, has been called in question; some asserting that the first sound is the result of the auricular contraction, and the second of the ventricular; some that they occur at the moment of the dilatation, not at the moment of the contraction of the cavities; and some that Laennec was right in regard to the ventricular sound, but that the second sound cannot arise from the contraction of the auricle, as Harvey, Haller, Senac, all declare that the auricle may be seen to contract immediately before the ventricular action, and they consider, therefore, the sound which follows the ventricular to be produced by some unknown cause, and the auricular contraction to be without sound,—two very singular and very considerable suppositions. The alteration of the sound in narrowing of the respective openings proves, I think, that Laennec is right. For if the opening from a ventricle is narrowed, the healthy sound ascribed by Laennec to the ventricles is altered; and if the opening from an auricle is narrowed, the healthy sound ascribed by Laennec to the auricle is altered. An argument in favour of the priority of the auricular contraction has been deduced from the veins of the neck in some cases regularly swelling immediately before the pulse is felt. But the obstruction in the auricles, causing this swelling, does not, I apprehend, occur during the contraction of the auricles; for at that moment there is a free space in the ventricles to receive the auricular blood, and it is only a part of the auricle that has the power of contraction. The obstruction which produces the swelling must take place as the ventricle becomes filled and the auricular blood consequently accumulates; and therefore the swelling of the veins must be expected when the ventricles will receive no more, viz. immediately before they contract, or while they are contracting. There is no wonder therefore that the arteries according to this account beat first: then a second sound of the heart is heard, I presume the auricular action; and then a short interval occurs before the veins pulsate —before the blood accumulates in the auricles previously to their contraction. The jugular veins are said by some, always to be dilated quite synchronously with the pulse of the arteries.*

From the occasional loudness of the morbid sound, wherever situated, (and I have sometimes heard it in various intensity at every part of the chest,) a difficulty sometimes occurs in deciding whether it originates on the left or right side. But a very careful examination of both halves of the cardiac region enables us in general to say distinctly that it is loudest on one side; and there we may always presume to be its source. Sometimes, but less frequently, a difficulty exists, from the extreme rapidity of the heart's action, as to whether the morbid sound

---

* "Mr. Adams, Dublin Hospital Reports, vol. iv. p. 426, 436.

"Since the delivery of these lectures, Laennec's accuracy has been called in question by others; and the stroke of the heart's apex, and the first sound of the heart declared to happen before the pulse, and to be produced by the dilatation and repletion of the ventricle, and the second sound to occur at the moment of the contraction of the ventricles, and to arise from the flapping of the parietes of the emptied ventricles together.

"I would reply in the first place, as before, that when an obstruction exists at the mouth of the aorta or pulmonary artery, a morbid sound occurs at the moment Laennec supposes the ventricles to contract; and when at either auriculo-ventricular opening, at the moment he supposes the auricles to contract. This could not happen had he mistaken the periods of the ventricular and the auricular contractions.

"Secondly, when the pulse at the wrist follows the stroke of the heart, it does so after only a very minute interval—such as may be explained by the distance of the radial artery from the heart, and actually occurs decidedly *before* the auricular sound—that which is now declared to be the ventricular. Moreover, when the pulse at the wrist is observed to follow the stroke of the heart, the pulse at the innominata (so much nearer the heart) may be found to precede that at the wrist, and to occur all but simultaneously with the heart's stroke, so that the relative distance of the parts explains the whole difference, and the pulsation of the arteries in all cases clearly arises from the stroke of the heart. If an artery is observed still nearer the heart than the innominata, no interval between its pulse and the stroke of the heart is perceptible. In four cases of aneurism of the ascending aorta, producing a strongly-pulsating tumour to the right of the sternum, this and the heart, when the fore-fingers were placed upon both, were felt, and by all seen, to pulsate quite synchronously. When the obstruction is at the mouth of the aorta or pulmonary artery, the preternatural sound I have always noticed synchronously with the pulse. When at an auriculo-ventricular opening, in the intervals of the pulse,—after or before the pulse. It sometimes in the latter case is so prolonged as to last till the pulse is again felt, so that there is no interval, but merely an equal alternation of the ventricular and the preternatural auricular sound: or even an interval occurs after the ventricular stroke, probably from the auricle not being disposed for contraction at the usual time, on account of its contraction having been so lengthened by the difficult escape of its blood that a longer repose is required than just during the ventricular contraction—here the auricular sound occurs first, then the ventricular, and then the interval.

"Thirdly,—the sounds considered by Laennec to be auricular and ventricular, are heard loudest, both in health and when morbid, at the seat of the auricles and ventricles respectively."

is heard at the ventricular or auricular contraction—as to whether the sound is heard at the moment of the arterial pulse or not. But by listening for a length of time and repeatedly during even only one visit, the difficulty generally vanishes; the confusion which at first prevailed declines; and the coincidence and succession of the phenomena become évident: exactly as happens, when, on first surveying a large collection of visible objects, all are confused, and at length every object is perceived distinctly, or when a person listens for the first time to the healthy sounds of the lungs or heart, and finds all confusion, but by perseverance gradually distinguishes each with perfect accuracy." 16.

Besides the preternatural sound, there is sometimes a thrill perceptible on applying the hand upon the region of the heart—a tremulous motion resembling the purring of a cat—most frequently dependant on narrowing of the auriculo-ventricular aperture of the left side.

"I believe also that, when this opening is narrowed, the pulse is generally irregular in both force and frequency,—a peculiar combination well known to practitioners, in which several beats succeed each other in rapid succession and with tolerable force, while others again occur slowly, and with little force, perhaps so weakly that they can scarcely or not at all be perceived, although at the heart the pulsation is evident enough. This most irregular pulse is thought by Mr. Adams to be pathognomonic of narrowing of the left auriculo-ventricular opening. Corvisart thought extreme irregularity of pulse in both force and frequency to characterise narrowing of the aortic opening. I do not happen to recollect a pulse irregular in force and frequency in a single case of narrowing of the aortic opening solely, while I know that it is very common in the narrowing of the left auriculo-ventricular opening, though possibly not peculiar to it, nor indeed to narrowing of any opening. Mr. Hodgson also mentions, that, in narrowing of the left auriculo-ventricular opening, there is often a double pulsation of the heart, *i. e.* two beats of the heart to one of the wrist—a pulsation of the heart from the action of the ventricle, and another from the action of the auricle. He appears to attempt to explain the fact, but this able surgeon expresses himself in rather an obscure manner. 'The auricle,' he says, 'first acts, and propels its contents into the ventricle; but, from the contraction of the communication the blood is not, as in the natural state, poured at once into that cavity. The ventricle, though imperfectly filled, contracts, and forces the blood into the aorta. Thus there is a pulse caused by the action of the auricle, and another by that of the ventricle, so that for every pulsation at the wrist, two are perceptible at the heart.' This does not appear to me a satisfactory explanation. The truth is, that in health there is a double action of the heart—one of the auricles and another of the ventricles—perceptible to the ear; though this was not generally noticed at the time Mr. Hodgson wrote. When the left auriculo-ventricular opening is narrowed, so that the blood can be only squirted into the ventricle, this auricular action is sometimes very distinct to the hand, in the condition of a thrill or purring, which is what Mr. Hodgson had remarked and called 'an irregular thrill (I use his own words), or *bruissement*, as Corvisart terms it, rather than a distinct pulsation.' Sometimes it is a distinct shock, and in another sentence Mr Hodgson speaks of it as such, saying, 'Thus there is a pulse caused by the action of the auricles, and another by that of the ventricles;' 'nor is the auricular pulse trifling.' This happens only, I believe, when the walls of an auricle are increased in substance—are in a state of hypertrophy, so that they act with a force approaching to that of the ventricles, and a double stroke of the heart may be felt by the hand. That this, and not the imperfect repletion of the left ventricle by which it acts twice, is the reason of the double stroke, appears to me shown by the fact that, when it has been observed, the auricle or auricles have always been hypertrophied, and in some cases the auriculo-ventricular opening not at all narrowed; and, secondly, that it is not observed, however narrow the auriculo-ventricular opening, if the auricle is not hypertrophied,—and, indeed, this hypertrophy is rare, as the obstruction usually produces dilatation and thinning." 17.

Another phenomenon resulting from narrowing of the left auricular opening is smallness of the pulse. The small quantity of blood thrown into the left ventricle at each auricular contraction may account for this diminution of pulse—but the same diminution takes place when the aortic aperture of the chamber is contracted, and the distinction can then only be ascertained by the sounds of the heart. Little indeed can be known by the smallness of the pulse alone. The size of the ventricular cavity may be so diminished as to produce a remarkably small pulse, of which we have given some curious examples in this Journal, from our own practice.

After a great many sensible observations on the *bruit de soufflet* and its causes, (which, by the way, are not yet clearly ascertained,) Dr. Elliotson goes to other points.

"Instances of obstruction at the mouth of the aorta are very common; indeed I am rarely without two or three such cases among my hospital patients, and I have never found this morbid appearance, where auscultation had not clearly shown its existence during life, by the constant preternatural sound at the moment of the ventricular action, and solely or most loudly in the region of the left ventricle.

Obstruction at the opening of the bicuspid or mitral valve is next in frequency, and I have never seen it, excepting once, where it had not during life produced some of these preternatural sounds constantly at the moment of the auricular contraction—in the intervals of the stroke of the heart, and solely or most loudly in the left half of the cardiac region—and at the cartilages of the left ribs, unless the left auricle and perhaps ventricle were dilated, when the upper part of the left half of the heart extends so much to the right, that the sound of the left auricle is often heard at the sternum or even still more to the right.

The right auriculo-ventricular opening I have never seen narrowed alone, and of the six instances of decided bellows-sound in the region of the right ventricle, and at the moment of the ventricular action, investigated by me after death, although two disclosed obstruction of the mouth of the pulmonary artery, as I have already stated, three occurred in the opposite state of the opposite opening in the ventricle—there was a permanent patency of the right auriculo-ventricular opening. The first instance occurred in a female twenty-seven years of age, who had suffered several attacks of acute rheumatism, the first of them eleven years previously, and for eight years had been subject to violent palpitation, dyspnœa, and pain in the region of the heart, with a sense of stricture and smarting in the front of the chest when supine, and of suffocation on exertion or mental emotion. The bellows-sound was heard in two parts of the cardiac region, and at the two periods of the heart's action. It was heard in the region of the left auricle, in the intervals of the stroke of the heart; and in the region of the right ventricle at the period of the ventricular action—at the moment of the pulse; and this constantly, for I examined her more than once after a long interval. The sound in the left and upper part of the cardiac region, at the moment of the auricular action, showed that the mitral or bicuspid valve was diseased, and the left auriculo-ventricular opening narrowed. There was a further confirmation of this opinion, if confirmation were necessary, in the circumstance of the pulse being small, though the heart acted violently and irregularly in strength and frequency. The bellows-sound in the right half of the cardiac region, and at the moment of the ventricular action, showed affection of one of the openings of the right ventricle. Never having read of a permanent patency of a cardiac opening and its effects, nor met with an example of it, I

hastily pronounced that, besides the narrowness of the mitral opening on the left side, there was an obstruction in the right ventricle at the mouth of the pulmonary artery. If permanent patency is a cause of bellows-sound, I ought to have only said affection of the right ventricle at one of its openings. For, on inspection, the course into the pulmonary artery was apparently free, but the tricuspid valve was bound down on one side, and therefore half useless, leaving a certain degree of passage to the blood of the right ventricle back into the right auricle when the ventricle contracted. The left ventricle was greatly hypertrophied and dilated, and the right auricle greatly dilated,—the former of which states had been indicated by the great force of the left ventricle, and the latter by the loud clear sound of the right auricle.—Very soon afterwards, I was desired to see a patient belonging to one of my colleagues, labouring under disease of the heart. Auscultation instantly showed hypertrophy of the left ventricle, and dilatation of the right auricle, and there was the bellows-sound in the right ventricle at the moment of the ventricular action. This last symptom caused me to expect disease at one of the openings of the right ventricle. The patient died the same day. The left ventricle was found hypertrophied, the right auricle dilated, and the tricuspid valve partially bound down, and therefore partly useless.—I witnessed a third instance of the bellows-sound in the right ventricle at the time of its action, in a negro, under the care of another of my colleagues; but the specimen, I believe, was not preserved. The left ventricle was hypertrophied, and the right auricle dilated. In these cases, the opening was not organically narrowed, nor the valve thickened, but simply bound down in half its extent. Had the valve been completely bound down, regurgitation would have been too free, I conceive, for the bellows-sound to have been produced. Playing partially, it left a narrow passage only back into the auricle.

A permanent patency of the tricuspid or mitral valves when these are thickened and their opening narrowed, is not uncommon. I doubt whether the semilunar valves of the aorta or pulmonary artery could be much bound down: they may be rendered partly useless, but are then always thickened, and ruptured, or their opening diminished. Neither, indeed, have I ever noticed the mitral valve to be bound down: though the bellows-sound in the left half of the heart, and disease of the mitral as well as of the semilunar valves, is of every day occurrence.

I do not recollect any allusion to this morbid state of the valves, except in the Annals of Medicine and Surgery, for 1816, in which a dissection is mentioned, and, among various morbid appearances, it is said that the tricuspid valve was partially bound down. Neither have I discovered in Laennec, Andral, Collin, or Bertin, any account of permanent patency from inutility of valves as a cause of the bellows-sound, except that Bertin describes a case of constricted mitral opening with great dilatation of the left auricle, in which there was a loud bellows-sound at the *auricular* action, and loudest in the region of the left auricle, as inevitably must have happened, but perceptible also in the region of the right auricle. The circumstance of the sound being perceptible in the region of the right auricle, he ascribes partly to the right auriculo-ventricular opening being so large that the tricuspid valve, which was diseased, was insufficient to close it. But he appears to me in error. It is very common to hear the bellows-sound, whether auricular or ventricular, in *both* halves of the cardiac region, when its source is in one only, at least in the left only. The sound, however, is always loudest in the half which is its source. And this extension of the sound is most remarkable when the cavity is dilated, the opening of which is diseased: for the cavity, at least the left, then encroaches upon the region of the other half of the heart. Now this actually was the fact in Dr. Bertin's case. The auricles were both dilated, but the left,—the seat of the bellows-sound, was dilated one-third more than the right. Besides, that he was wrong is farther shown by the bellows-sound occurring at the *auricular* contraction on the right side no less than on the left where its source was the narrowness of the left auriculo-ventricular opening. Had the permanent patency of the right auriculo-ventricular opening caused the extension of the sound to the right side, it would have been heard at the *ventricular* contraction, for the inutility of the valve would have operated at no other time—could have made no difference to the course of the blood from the auricle.—The opinion of the permanent patency of a cardiac opening from any cause, as a source of the bellows-sound, I heard first from Dr. James Johnson. Who originally suspected it, I cannot say. Dr. Johnson imagined he had learnt it from Laennec and other writers upon auscultation ; but, as I have already stated, I have found no other notice of it than the erroneous view of Bertin. Preternatural patency sometimes arises from dilatation of the cavity on either side, and enlargement of the opening, so that the valve though healthy, has become insufficient to close it entirely, leaving a narrow passage for regurgitation.*—I may mention that a relative narrowness of an opening occasions the *bruit de soufflet* as well as an absolute narrowness—*i. e.* the size of the opening remaining the same, but the cavity behind it becoming dilated, and its walls perhaps thickened, the natural proportion between the two is lost, and the effects of real and absolute constriction are produced. This I think I have seen in more than one case of dilatation and hypertrophy of the left ventricle.—The silence of authors upon the morbid patency of the cardiac openings from the adhesion of the valves, except in the one case in the London Annals of Medicine and Surgery by Dr. Carbut of Manchester, inclines me to believe that many cases in which the bellows-sound had been heard, and yet no morbid appearance was discovered to explain it, were probably cases of morbid patency which was entirely overlooked ; more especially when I reflect that no fewer than three cases of this kind have occurred to me during the last few months.

Still in all the three cases in which the tricuspid was bound down, and the bellows-sound had been heard, there was hypertrophy or dilatation, or both, in some compartment of the heart, and this might probably have so affected the opening of the cavity at which the sound was heard, as to contribute to the production of the sound : in all three the left ventricle was hypertrophied and dilated, and the right auricle greatly dilated. This is further probable from the fact, that, when the tricuspid has not been bound down, and there was hypertrophy or dilatation or both, in the left side of the heart, or dilatation of the right auricle, I have frequently heard the bellows-sound, auricular or ventricular : and yet, although I cannot imagine the bellows-sound to have any other than a mechanical cause, the relaxed state of the parts after death allowed no affection of the opening of the auricle or ventricle to be discoverable. And further, to prove that the cause was mechanical, a purring thrill was *felt* by the hands, in some of these very instances, at the moment of the *sound*.

Another source of the many instances of declared absence of morbid appearance to explain previous bellows-sound is, I am convinced, the custom of slitting up the openings, especially the ventriculo-arterial, without previously passing the fingers through to ascertain whether any constriction exists. For this sometimes occurs at the root of the valves, though their appearance is perfectly healthy ; and the slightest diminution of an opening, whether from internal causes or external pressure, is sufficient to induce a degree of bellows-sound,

---

* See, for instance, Dr. Abercrombie's Paper on the Pathology of the Heart, in the Edinburgh Medico-Chirurgical Transactions, Vol. I. Case 16 and 17. In Case 17, corrugation of the valve and morbid enlargement of the opening conspired.

In all three the auricle was greatly dilated, and in one the ventricle also.

such as may readily escape detection and not have produced any marked derangement of circulation or respiration.

It is also to be remembered, that those who are not familiar with the diseases of the heart, very easily pass over a morbid appearance, just as those who have not spent some time upon auscultation, pass over the most morbid sounds during life, although they listen.

I should be inclined to consider this morbid patency of the tricuspid valve, or some change of the opening of the right ventricle by the hypertrophy or dilatation of some part of the heart, the most frequent cause of the bellows-sound in the right ventricle. For thickening and narrowing of the valves on the right side of the heart are comparatively so rare that Bichat declared he never saw disease in them. Bertin, in the course of twenty years met with important induration in the tricuspid, even of a cartilaginous character, but four times. Disease of the mouth of the pulmonary artery is still rarer. A case is mentioned by Bertin where a sort of hymen existed at the mouth of the pulmonary artery; but it was probably congenital, and this is the only instance of obstruction that he ever saw at that spot. Dr. Hue has just presented to the College an extraordinary heart, taken from a patient in St. Bartholomew's Hospital. The walls of the right ventricle appear grown up around the mouth of the pulmonary artery, so that the opening from the ventricle is no larger than the circumference of a goose-quill; and a short canal of this dimension had to be traversed by the blood before it reached the mouth of the pulmonary artery, which is of the usual size. This state was probably, like Bertin's case, congenital, for two additional and evidently congenital cavities exist in the substance of the heart—two supplementary little right ventricles,* one leading from the pulmonary artery, and the second from the first. A bellows-sound occurred in the heart, with venous congestion and dropsy. I have never seen obstruction from disease of the pulmonary semilunar valves. Bertin refers to two, one in Morgagni, and one seen by M. Louis at la Charité. But in both, as well as in that which he himself saw and which was just alluded to, both halves of the heart communicated, so that the right was in the condition of the left—washed by a portion of arterial blood, and it is under such circumstances that ossification or extensive cartilagination of the valves of the right half takes place. I have but once seen material induration of the tricuspid valve and narrowing of its opening; three times only impediment, visible after death, to the entrance of the blood into the pulmonary artery, and then, as I have said, not from any disease of the valves, but in two of them from the accidental situation of a piece of cartilage in the substance of the heart near the commencement of the artery, and in the third, Dr. Hue's case, from the right ventricle being grown together just before the origin of the artery. Yet I have three times seen the tricuspid partially bound down, without being thickened or otherwise diseased; the right auricle however being dilated. I have heard a bellows-sound at both sides, and at the ventricular action, from a piece of bone lying between the pulmonary artery and aorta, and very slightly compressing both; and several times I have heard it constantly at the ventricular action, in patients who died of disease of the left side of the heart, with enormous *dilatation of the right auricle which appeared necessarily to have compressed the aorta*, and in some even the pulmonary artery, especially in the supine posture. An increased thickness of the muscular substance, near an opening, without any disease of the valves, will cause impediment enough for a degree of the preternatural sound, though the diminution of the opening is not appreciable to any but an experienced eye." 21.

* On Supernumerary Parts of the Heart, see Andral, Précis d'Anatomie Pathologique, T. II. p. 313, sq.

Dr. E. remarks, and the remark is important, that, "as the openings of the veins into the auricles are unprovided with valves, no morbid patency of them can give rise to an *auricular bellows-sound.*" Now the bellows-sound takes place at the same instant that the heart strikes against the side, as any person may convince himself of, who has a patient presenting the *bruit de soufflet*—and consequently this pathological fact negatives the new theory of the stroke against the ribs being produced during the dilatation of the ventricles. But we must bring this notice of Dr. Elliotson's second lecture to a conclusion, with the following extract.

"Besides these, the general symptoms of obstruction in the heart are dyspnœa, perhaps amounting to orthopnœa, palpitation, quickness, and intermittence, of the heart's action, starting up from sleep, paleness and leaden hue of the countenance, blueness of the lips and tongue, suffusion of the eyes, fulness of the under eyelid, anasarcous swelling of the face, and indeed of the whole body, generally commencing in the feet, paucity of urine, distention and pulsation of the jugular veins, flatulence, &c. The dyspnœa is usually greater when the disease is upon the left side, but chronic inflammation of the lungs, *i. e.* chronic bronchitis, so generally accompanies disease of every portion of the heart, and indeed is so common a cause of death in all cardiac diseases, that the dyspnœa is sometimes intense in obstruction on the right side. The distention of the veins is usually more considerable when the obstruction is on the right side; great obstruction, however, on the left side has the same effect, and so likewise has mere congestion or chronic inflammation of the lungs.

But these general signs are insufficient to point out the seat of the obstruction, and often indeed its existence, for they accompany all other kinds of diseases of the heart, and even many affections of the lungs, and the collection of fluid in the pleuræ; and I know that without the aid of the peculiar sounds which have been discussed, the most erroneous diagnoses are every day made. Long before any other symptom arises from the obstructing cause, these preternatural sounds indicate its existence, and point out cardiac affection which might not have been suspected till irremediable.

Disease of the valves generally produces or is occasioned by disease of the substance of the heart itself, so that other cardiac symptoms, as well as the preternatural sound, present themselves." 22.

In another article we shall present our readers with an analysis of Dr. E.'s third and concluding lecture.

